# Evaluation of three different rotary systems during endodontic retreatment - Analysis by scanning electron microscopy

**DOI:** 10.4317/jced.52685

**Published:** 2016-04-01

**Authors:** Flávia-Teixeira Vidal, Eduardo Nunes, Martinho-Campolina-Rebello Horta, Maria-Rita-Lopes-da Silva Freitas, Frank-Ferreira Silveira

**Affiliations:** 1 DDS, MS, Graduate student, Departament of Dentistry, Pontifícia Universidade Católica de Minas Gerais, Belo Horizonte, Brasil; 2 DDS, MS, PhD, Professor, Departament of Dentistry, Pontifícia Universidade Católica de Minas Gerais, Belo Horizonte, Brasil; 3 DDS, MS, Professor, Departament of Dentistry, Pontifícia Universidade Católica de Minas Gerais, Belo Horizonte, Brasil

## Abstract

**Background:**

Endodontic therapy is considered a series of important and interdependent steps, and failure of any of these steps may compromise the treatment outcome. This study aimed to evaluate the effectiveness of three different rotary systems in removing obturation materials during endodontic retreatment using scanning electron microscopy (SEM) analysis.

**Material and Methods:**

Thirty-six endodontically treated teeth were selected and divided into 3 groups of 10 and 1 control group with 6 dental elements. The groups were divided according to the rotary system used for removing gutta-percha, as follows: G1: ProTaper system; G2: K3 system; G3: Mtwo system; and G4: Control group. Thereafter, the roots were split and the sections were observed under SEM, for analysis and counting of clear dentinal tubules, creating the variable “degree of dentinal tubule patency” (0: intensely clear; 1: moderately clear; 2: slightly clear; 3: completely blocked). The data were subjected to the Friedman and Kruskal-Wallis statistical tests.

**Results:**

No differences were observed in the “degree of dentinal tubule patency” neither between the root thirds (to each evaluated group) nor between the groups (to each evaluated third). Nevertheless, when the three root thirds were grouped (providing evaluation of all root extension), the “degree of dentinal tubule patency” was lower in G1 than in G3 (*p*<0.05), but showed no differences neither between G1 and G2 nor G2 and G3.

**Conclusions:**

No technique was able to completely remove the canal obturation material, despite G1 having shown better results, although without significant difference to G2

** Key words:**Scanning electron microscopy, NiTi, retreatment.

## Introduction

The basic objectives of endodontic treatment are cleaning and shaping of the root canal system, as well as its three-dimensional obturation. Adequate obturation must provide hermetic sealing, prevent re-infection, and promote the biological repair process of periapical tissue (1).

In seeking a new, high-quality intervention, several studies have been conducted to find a more suitable, effective, and rapid working system that does not interfere with biological aspects and allows access throughout the length of the root canal. Even with the most modern techniques, failures in endodontic treatment are sometimes unavoidable, and it is often necessary to remove all of the obturation materials used to restore the healthy periapical tissue (2).

The aim of this study was to evaluate the efficiency of different rotary instrument techniques in removing root canal obturation material, analyzing clear dentinal tubules in the cervical, middle, and apical thirds.

## Material and Methods

After review and approval by the Pontifícia Universidade Católica de Minas Gerais (PUC Minas) Ethics Committee, 36 extracted single-rooted human teeth were selected for this study. The dental elements were endodontically treated during pre-clinical training in Endodontics at PUC Minas with the same protocol, using gutta-percha # 30 master cone, lateral condensation technique and endodontic sealer.

All specimens were radiographed using a digital radiography system (VISTEO T2, Owandy, France) and the selected teeth displayed good obturation according to the criteria of Santos *et al.*, 2010 (3).

The teeth were divided into 3 groups of 10. The length of the teeth was standardized to 21 mm by erosion of the incisal and/or occlusal surface with a caliper rule (Starrett Indústria e Comércio LTDA, São Paulo, Brazil) and a 1557 drill (KG Sorensen, São Paulo, Brazil) at high speed. The procedure for removing the root canal obturation material was started in both groups.

In Group I (G1), the removal procedure was performed with ProTaper Universal Retreatment rotary files (DentsplyMaillefer, Ballaigues, Switzerland). The instruments were coupled to a 16:1 speed-reducing contra-angle handpiece and used with an XSmart electric motor (Dentsply-Maillefer, Ballaigues, Switzerland). The speed was 300 rpm, and the torque was 3 N.cm, as per the manufacturer’s instructions. D1 files (30.09) were used for filling removal in the cervical third; D2 files (25.08) were used for filling removal in the middle third; and D3 files (20.07) were used for filling removal in the apical third. An F4 finishing file was then used to complete the root canal preparation.

In Group II (G2), K3 system files were used (Sybron Dental Specialties, Kerr Corporation, Orange, CA, USA). The nickel-titanium (NiTi) retreatment instruments were employed in the following sequence: 40/0.06 taper, 35/0.04 taper, and 30/0.04 taper in the cervical, middle, and apical thirds, respectively, and ending with a 35/0.04 taper. The speed used was 300 rpm, with 1.2 N.cm torque as per the manufacturer’s instructions. An XSmart electric motor was used.

In Group III (G3), retreatment was performed using an Mtwo Rotary System (VDW, Munich, Germany). The Mtwo System R2 instrument (25.05) was used at a speed of 300 rpm and torque of 1.2 N.cm, as per the manufacturer’s instructions, in the cervical, middle, and apical thirds; an XSmart electric motor was used. Preparation was completed with an Mtwo 40.05 file.

In Group IV (G4) specimens were not retreated (control group).

During the retreatment, irrigation was performed in all groups, using 1 mL of 2.5% NaOCl (Lenzafarm, Belo Horizonte, Brazil) between each instrument; final irrigation was performed with 3 mL of a 17% EDTA solution (Biodinâmica Ibiporã, Paraná, Brazil) for 5 minutes with the aid of an Irrisonic tip (Helse Ind. e Comércio LTDA, Sorocaba, Brazil) attached to a Jet Sonic ultrasonic device (Gnatus, São Paulo, Brazil) for 30 seconds at power level 1. Final irrigation was performed with 1 mL of 2.5% NaOCl.

Each instrument was used up to five times and then discarded at any sign of deformation. All instruments were used in a back-and-forth motion with gentle apical pressure and movements against the canal walls.

After filling removal, the three groups of teeth were radiographed using a digital system (VISTEO T2, Owandy, France) to document the procedures performed.

After retreatment (and additionally for the control group specimens), the teeth were grooved with a diamond saw and split longitudinally using chisel and mallet. The half that had the largest amount of visible gutta percha was selected and placed under the JSM-6510 LV Scanning Electron Microscope (Jeol, Tokyo, Japan), mounted on an SEM support, after gold plating.

To analyze the presence of clear dentinal tubules in the apical, middle and cervical thirds of the three groups, areas centrally located 5, 10 and 15 mm from the apex were marked and analyzed in all dental elements at 2,000x magnification.

Two calibrated evaluators counted the number of clear tubules on the selected images. The scores used were classified as follows (creating the variable “degree of dentinal tubule patency”): 

0): intensely clear tubules; more than 20 clear dentinal tubules;

1): moderately clear tubules; 11 to 20 clear tubules;

2): slightly clear tubules; up to 10 clear tubules;

3): tubules completely blocked; no dentinal tubule visibly clear.

The Friedman test followed by Dunn’s post hoc test was used to evaluate the existence of differences in the “degree of dentinal tubule patency” variable between each root third in each of the three study groups.

The Kruskal-Wallis test followed by Dunn’s post hoc test was used to evaluate the existence of differences in the variable “degree of dentinal tubule patency” among the three evaluated groups, separately for each of the thirds and with the three thirds grouped. The level of significance was set at 5%. The analyses were performed using the GraphPad Prism Software (GraphPad Software, San Diego, California, USA).

## Results

**Figure 1:**
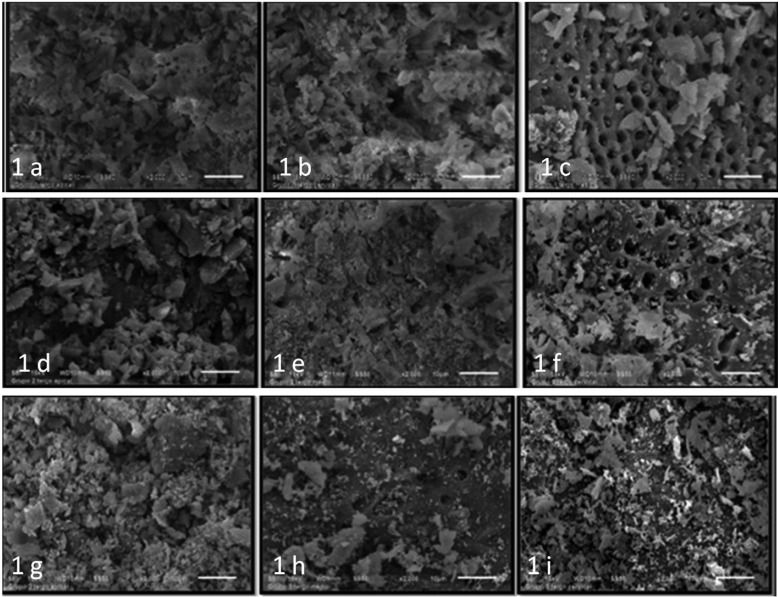
Representative SEM images from G1 (a - apical, b - middle and c - cervical), G2 (d - apical, e - middle and f - cervical) and G3 (g - apical, h - middle and i - cervical).

Figure 1 shows representative SEM images from G1 (a-c), G2 (d-f) and G3 (g-i).

The Friedman test showed no differences in the “degree of dentinal tubule patency” variable among each root third in each of the three study groups (*p*>0.05) ( Table 1).

**Table 1:**
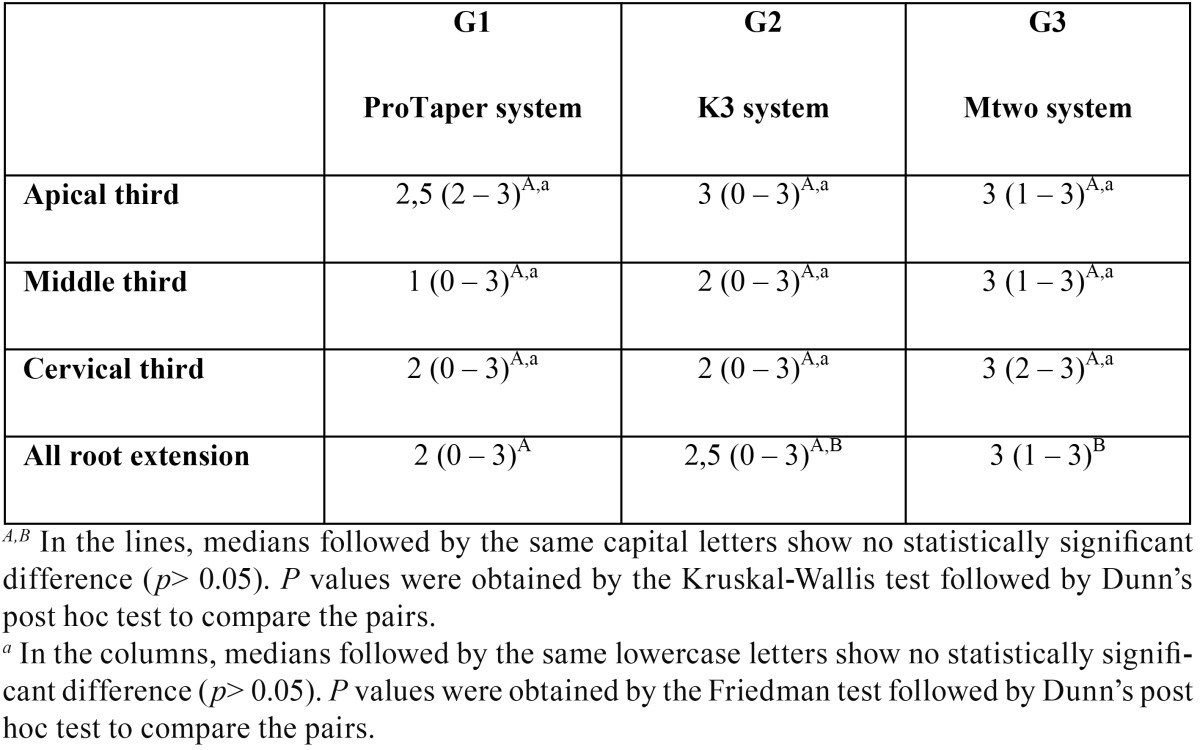
Median, minimum and maximum value of the variable “degree of dentinal tubule patency”.

The Kruskal-Wallis test revealed no differences in the variable “degree of dentinal tubule patency” among the three groups when the thirds were evaluated separately (*p*>0.05) ( Table 1).

The Kruskal-Wallis test showed the presence of differences in the “degree of dentinal tubule patency” variable among the three groups when the thirds were grouped. Dunn’s post hoc test revealed a significant difference when comparing G1 and G3 (*p*<0.05) but not when G1 and G2 (*p*>0.05) and G2 and G3 (*p*>0.05) were compared ( Table 1).

In summary, no differences were observed in the “degree of dentinal tubule patency” neither between the root thirds (to each evaluated group) nor between the groups (to each evaluated third). Nevertheless, when the three root thirds were grouped (providing evaluation of all root extension), the “degree of dentinal tubule patency” was lower in G1 than in G3 (*p*<0.05), but showed no differences neither between G1 and G2 nor G2 and G3.

## Discussion

During the present study and in order to eliminate possible interfering factors, we had the concern to carry out standard procedures and performed by a single operator. The use of nickel-titanium instruments, electric motor with a low torque, was established according to the manufacturer’s protocol. These conditions can contribute to increased touch sensitivity and prevent the instruments from fracturing (4). The teeth were treated endodontically by undergraduate students at the PUC Minas Department of Dentistry, who followed the same preparation protocol and filling, to simulate clinical reality as closely as possible.

Different methods have been employed to evaluate the efficacy of endodontic retreatment. Optical microscopy has been used to evaluate the removal of remaining obturation material (5). Computed tomography is another method that has been employed to evaluate the percentage of remaining obturation material in endodontic retreatment performed with manual and rotary NiTi instrumentation (2). In the present study, a scanning electron microscope was chosen to obtain images of the root canal after obturation material removal, with a considerable magnification of 2,000x. This method enables the remaining obturation material and dentinal tubules to be observed and identified through the acquisition of high resolution images (6).

The effectiveness of retreatment with rotary instruments has been confirmed in many studies (7-9). These techniques have been proposed as an alternative to manual instrumentation. However, manual techniques should be considered, as some studies still demonstrate cleaner walls when using this technique. Others investigations, however, reveal no difference in material removal between manual and rotary files (2-10).

In all root canals used in this study, obturation material residue could be observed after endodontic retreatment regardless of the root third, indicating that such residues were a constant across all systems. These data are consistent with previous studies that investigated different methods of removing obturation material in retreatment cases and revealed that no technique can fully remove gutta-percha and sealer from the inside of root canals (4,5,11,12).

Most of the reports do not detect differences in techniques for total area of values gutta-percha remotion. The explanation for this can be credited to not use solvents. However, the use of solvent to reach the working length and remove the gutta-percha appears to have no influence on the filling material removal capacity (4,11). Still, as the goal of the study was to investigate the mechanical removal capacity of filling material through rotary systems, no solvent was employed. A general analysis of the results revealed that there is a need to improve the rotary technique removal of filling material. It is noteworthy that indicated endodontic retreatment, there is the need to remove the waste material, as this can provide free from microorganisms, and hinder the mechanical action of the instruments as well as the chemical action of auxiliary substances to the preparation of the root canals. Although contradictory in the literature, in the present study was used an irrigation protocol, using little inserts adapted to ultrasound, this maneuver aimed at increasing the effectiveness of the action of irrigating solutions (13-15). Manual techniques should be considered, as no technique can separately fully effective removal of filling materials. Therefore, the association with manual techniques and the use of solvents, optical microscopy and Gates Glidden drills use may be important to maximize the success of an endodontic retreatment.

Based on our methods and results, it is possible to conclude the following: 1) none of the techniques used had the ability to completely remove obturation material from the root canals; 2) comparing the three groups, G1 (ProTaper system) had better results without, although significant difference to G2.
